# Analysing cause of death during follow-up for non-muscle-invasive bladder cancer: is there a role for watchful waiting?

**DOI:** 10.1308/rcsann.2022.0099

**Published:** 2022-10-14

**Authors:** Y Zhang, M Nosseir, J Dyer

**Affiliations:** Stockport NHS Foundation Trust, UK

**Keywords:** Bladder cancer, Frailty, Survival, Watchful waiting

## Abstract

**Introduction:**

Non-muscle-invasive bladder cancer (NMIBC) patients often require multiple invasive procedures during follow-up. Surveillance guidelines do not adjust for increasing frailty or competing comorbidity. We aim to evaluate the influence of these factors on the natural history of NMIBC and whether this may have implications for appropriate follow-up schedules.

**Methods:**

NMIBC patients who died in a 3-year period while on cystoscopic surveillance were identified. Frailty was assessed using the Rockwood Clinical Frailty Scale (CFS): 1–3, no frailty; 4, vulnerable; 5–9, mild/severe frailty. Similarly, three-tier categorisations were performed for comorbidity (Charlson Comorbidity Index) and for anaesthetic risk (American Society of Anesthesiologists’ [ASA] score).

**Results:**

Of the 69 patients, 26 were categorised as no frailty, 20 as vulnerable and 13 as frail. There was no difference in the proportions of those with higher risk NMIBC between the categories. Increasing frailty was associated with reduced overall survival (median 59, 29 and 13 months; *p *< 0.05) but not recurrence-free survival (*p *= 0.98) or progression-free survival (*p *= 0.58). Similar results were obtained using the Charlson Comorbidity Index or ASA score. No frail patients with low/intermediate-risk NMIBC had clinically significant disease progression prior to death. Frail patients with CFS ≥ 4 were found to have similar complications due to bladder cancer itself (*p *= 0.48) yet almost three times as many complications following cystoscopic procedures during follow-up (*p *< 0.05).

**Conclusions:**

For frail patients with low risk of progression, protocol-driven cystoscopic surveillance may not improve survival and watchful waiting may be more appropriate. Further investigation is required to determine the feasibility of this approach.

## Introduction

The majority of new UK bladder cancer diagnoses are made in people aged over 75 years, with the highest incidence in the 80–85 years age group.^1^ Some 75% of bladder cancers are diagnosed as non-muscle-invasive (NMIBC). Treatment and follow-up depend on various prognostic factors such as patient age, tumour size, multiplicity, stage, grade, recurrence rate and histological variance.^2^

NMIBC has a strong tendency to recur so there exists a period of risk-stratified cystoscopic surveillance of varying intensity after initial treatment with transurethral resection of bladder tumour (TURBT). Flexible cystoscopy itself has been shown to be a painful experience for a large proportion of patients.^3^ There are also significant financial and service costs to health providers, with bladder cancer having the highest lifetime treatment cost per patient of all cancers.^4^

Frailty is a clinical syndrome characterised by increased vulnerability resulting from ageing-associated physiological decline across many systems and the inability to cope with everyday acute stressors.^5^ In addition to chronological age, the Charlson Comorbidity (Ch) Index^6^ and American Society of Anesthesiologists (ASA) physical status score^7^ are often used as proxies for frailty, but they are not synonymous.

Rockwood’s nine-point Clinical Frailty Scale (CFS) is a compromise between a simple test that can be used routinely in the clinical setting and one that is sensitive enough to detect small differences in frailty.^8^ Frailty is of particular interest to surgeons because it is an independent risk factor for major morbidity, mortality, protracted length of stay and institutional discharge^9^ across multiple care settings.^10,11^ Furthermore, the CFS has been validated both prospectively^12,13^ and retrospectively^14,15^ when used by junior clinical staff outside the geriatric setting.

Watchful waiting is a joint decision to delay treatment of a disease until the symptoms become significant with the aim of not altering disease-specific survival. It is commonly used in the setting of prostate cancer.^16^

There are risk nomograms for disease recurrence and progression for NMIBC, but no national or international guidance advocating watchful waiting for certain NMIBC patients. The European Association of Urology (EAU) asks that low-risk patients are followed-up for 5 years and high-risk patients for life, with intermediate-risk patients having an ‘in-between’ follow-up scheme.^2^ National Institute for Health and Care Excellence (NICE) guidelines suggest low- and intermediate-risk patients are followed-up for 1 and 5 years, respectively.^17^ Our primary aim is to study the natural history of NMIBC in patients of varying frailty with the goal of determining whether there is justification for a watchful waiting cohort. Our secondary aim is to study how different Charlson and ASA scores associate with the natural history of NMIBC.

## Methods

Suitable patients from a single institution were identified from clinical coding. All patients had died during a 3-year period between 1 January 2017 and 31 December 2019 with a diagnosis of bladder cancer and had undergone flexible cystoscopy within the 2 years prior to death. The maximum interval between cystoscopies is approximately 12 months so we inferred that this should capture all patients on surveillance for NMIBC. Relevant clinical information was obtained from electronic patient records. Where key information was lacking, paper notes were interrogated. Patients were excluded if they had an index diagnosis of muscle-invasive bladder cancer or if they previously had upper tract urothelial cancer. Similarly, patients who did not have full records of all histological analyses were also excluded; this was usually the case for index diagnoses made prior to electronic documentation (circa 2004) and/or in different hospital trusts.

Patients were added to the Stockport bladder cancer database, an electronic record of local patients who have had or are on surveillance for NMIBC. This resource was established in 2018 and maintained prospectively. It aims to interrogate the data for information about the natural history of the disease as well as to record and analyse local practice. Data were recorded using Microsoft Access (Microsoft Corporation, 2010).^18^ Features of the index TURBT were recorded including date of operation, and size and number of lesions. Tumour histology was recorded as per TNM staging and World Health Organization grading (1973 and 2004 versions).^2^ All subsequent surveillance cystoscopy dates and findings were recorded. Any interventions required from these were also documented in a manner similar to the index TURBT.

Progression was defined as per the International Bladder Cancer Group’s recommendation for NMIBC.^19^ Recurrence was confirmed either by histological analysis or visual confirmation prior to fulguration when a specimen was not obtained. Patients who died were categorised as a bladder cancer death or non-bladder cancer death based on the death certificate and/or electronic hospital notes where available. For patients who died outside hospital or where the relevant documentation was not available to review, a consultant urologist reviewed the patient’s case and categorised accordingly.

Frailty assessment was performed using the nine-point CFS^20^ based on the patient’s preoperative assessment at their index TURBT. Similarly, comorbidity burden using the Charlson index and anaesthetic risk via the ASA score were taken at this time. When there was not enough information to confidently score a patient, a consultant urologist’s opinion was sought. If the latter did not yield a score, then the data point was left blank. We stratified these scores into three categories for analysis. CFS scores were divided into ≤3 (not frail), 4 (vulnerable) and ≥5 (frail) as per their nine-point CFS definitions. ASA scores were divided into ≤2 (mild systemic disease), 3 (severe systemic disease) and ≥4 (life-threatening systemic disease). Charlson indices were divided into ≤5, 6–7 and ≥8. All study patients scored for age and a further two points for having a solid tumour. Patients with CFS ≤3, Charlson ≤5 and ASA ≤2 were used as reference categories for comparison.

The rates of surveillance flexible cystoscopies and non-surveillance procedures were extrapolated based on the number of these during individual follow-up periods and expressed as number of procedures per year.

Statistical analysis was performed by the authors. Kaplan–Meier was used to analyse overall survival (OS), recurrence-free survival (RFS) and progression-free survival (PFS). This was performed on RStudio (2021).^21^ Student’s *t* test (assuming unequal variances) was used to compare the reference category against the other remaining two categories. This was done for rates of surveillance flexible cystoscopies and non-surveillance procedures. Fisher’s exact test was used to check for significance between frailty groups with respect to bladder cancer mortality and/or progression and EAU NMIBC risk categories^14^ (Tables 1 and 2). Significance was accepted when *p* < 0.05.

**Table 1:** Demographics and characteristics of the study population

	CFS			Charlson			ASA		
	≤3	4	≥5	≤5	6–7	≥8	≤2	3	≥4
Number, *n*	37	19	13	21	30	18	21	31	9
Mean age, years	76.2	78.7	82.3	71.6	79.4	83.4	76.1	79.4	79.4
Median age, years	78.1	80.0	84.3	70.5	80.2	84.1	78.0	81.8	78.8
Age standard deviation, years	9.08	7.62	8.81	6.99	9.03	5.55	8.67	8.84	9.21
Male sex, %	78.4	73.7	76.9	71.4	80.0	77.8	75.9	77.4	77.8
Higher risk, %	59.5	47.4	69.2	57.1	56.7	61.1	48.3	61.3	77.8
	ref	(0.411)	(0.742)	ref	(1)	(1)	ref	(0.437)	(0.148)

The total study population (N = 69) was categorised by way of the Rockwood nine-point Clinical Frailty Scale (CFS), Charlson Comorbidity Index and American Society of Anesthesiologists (ASA) score. ‘Higher risk’ non-muscle-invasive bladder cancer is defined as European Association of Urologists ‘high’ or ‘very high’ risk categories.^13^ Comparisons were made between the healthiest reference (ref) categories against the others; *p*-values (in parentheses) were calculated using Fisher’s exact test.

**Table 2:** The natural history of NMIBC and follow-up regimes compared across the study groups

	CFS			Charlson			ASA		
	≤3	4	≥5	≤5	6–7	≥8	≤2	3	≥4
Overall survival, years	58	25	13	59	38.5	20	60	30	21
	(**<0.01**)			(**<0.01**)			(**<0.01**)		
Recurrence-free survival, years	24	16	27	26	21	25	26	24	16
	(0.98)			(0.76)			(0.53)		
Progression-free survival, years	144	n/a	n/a	144	n/a	n/a	n/a	144	n/a
	(0.58)			(0.39)			(0.65)		
Surveillance, per year	1.73	0.31	1.08	2.00	1.32	1.08	1.78	1.16	1.47
	ref	(**<0.01**)	(0.105)	ref	(0.206)	(0.112)	ref	(0.128)	(0.563)
Non-surveillance, per year	0.536	0.404	0.261	0.664	0.370	0.325	0.430	0.525	0.240
	ref	(0.323)	(0.102)	ref	(**0**.**0263**)	(**0**.**0278**)	ref	(0.432)	(0.307)
BCa death, %	18.9	5.3	15.4	28.6	10.0	5.6	20.7	9.68	11.1
	ref	(0.243)	(1)	ref	(0.136)	(0.0985)	ref	(0.437)	(0.148)
BCa death/Prog, %	29.7	15.8	15.4	33.3	20.0	16.7	20.7	25.8	22.2
	ref	(0.338)	(0.469)	ref	(0.338)	(0.290)	ref	(0.763)	(1)

Median overall survival, recurrence-free survival and progression-free survival are shown in years. The rates of surveillance and non-surveillance cystoscopies are shown per year. The proportions of bladder cancer (‘BCa’) deaths and/or progressions (‘prog’) are shown. Comparisons were made between the reference (ref) categories against the others. Survival analysis performed using Kaplan–Meier for overall, recurrence-free and progression-free survival with associated *p*-values is shown. *p*-values (in parentheses) were calculated using Student’s *t* test for continuous variables and Fisher’s exact test for categorical variables.

ASA = American Society of Anesthesiologists; CFS = Rockwood Clinical Frailty Scale; n/a = median not defined.

The proportion of patients who had a bladder cancer recurrence as well as those who progressed or died from bladder cancer were analysed in two-by-two matrices with two combined frailty groups: non-frail (CFS ≤ 3) vs frail (CFS ≥ 4) as well as between two combined EAU NMIBC risk groups: low/intermediate vs high/very high.

Case notes were also reviewed to tally the number of complications within 30 days of a bladder cancer procedure (surveillance or non-surveillance). They were divided into complications directly attributable to the procedure and those that were unrelated but still occurred within 30 days. These complications were expressed as rate per procedure and Fisher’s exact test was used to compare groups. Acute admissions during follow-up that were attributable to complications of the natural history of bladder cancer (e.g. haematuria, bladder infection or upper tract obstruction) were also counted and expressed as a mean per patient; Student’s *t* test was used to compare groups here. When there was ambiguity regarding the nature of an admission, a consultant urologist’s opinion was sought.

## Results

Sixty-nine patients were identified with a mean age of 78.0 years (range 50.2–94.1) and 76.8% were male. The risk profile as per the EAU nomogram was as follows: low, 20.3%; intermediate, 21.7%; high, 52.2%; and very high, 5.80% (Table 1). Median OS was 32 months.

**Figure 1:**
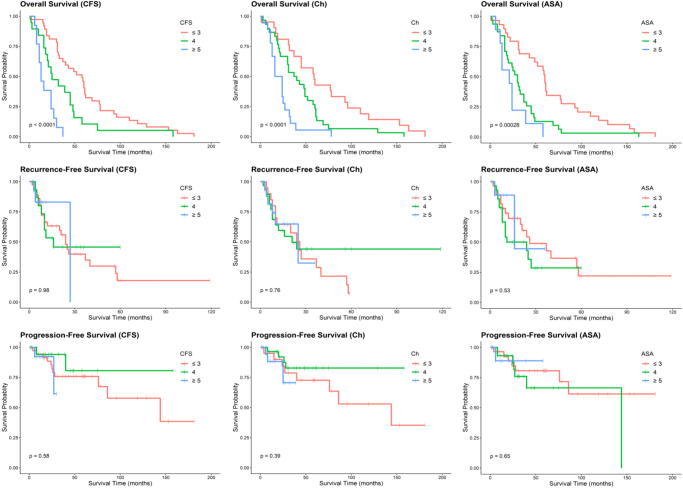
Kaplan–Meier analyses comparing the Rockwood Clinical Frailty Scale (CFS), Charlson Comorbidity Index (Ch) and American Society of Anesthesiologists (ASA) physical status score categories with respect to overall survival (OS), recurrence-free survival (RFS) and progression-free survival (PFS). Survival time is shown in months. Hashed marks represent censored data. *p*-values are shown for each analysis.

The patients were divided into three categories according to CFS, Charlson and ASA scores. For all these parameters, increasing frailty, comorbidity and anaesthetic risk were associated with reduced OS (Table 2). Conversely, there was no statistical significance between categories when RFS or PFS was analysed (Table 2). This relationship is shown in the Kaplan–Meier plot (Figure 1).

There was no difference between categories in the rate of flexible cystoscopies, except for fewer cystoscopies in the ‘vulnerable’ (CFS = 4) group when compared with the reference cohort (Table 2).

Similarly, there was no appreciable difference in the rate of non-surveillance procedures between CFS and ASA categories. There was, however, a significant difference when comparing higher Charlson scores (more comorbidity) with the reference cohort (Table 2).

There was no statistical difference in bladder cancer death and/or progression and the proportion of high (including EAU ‘very high’) risk index tumours between the categories (Table 3).

**Table 3:** Two-by-two matrices showing the proportion of patients who died/progressed of bladder cancer as well as those who recurred

	Bladder cancer death/progression		Bladder cancer recurrence	
	Lower risk	Higher risk	Lower risk	Higher risk
Non-frail	2/16 (12.5%)	10/21 (47.6%)	11/16 (68.8%)	14/21 (66.7%)
Frail	1/13 (7.69%)	3/19 (15.8%)	2/13 (15.4%)	9/19 (47.4%)

Non-frail (CFS ≤ 3) and frail (CFS ≥ 3) patients were compared with lower (low/intermediate) and higher (high/very high) risk NMIBC as defined by the EAU.^13^

CFS = Rockwood Clinical Frailty Scale; EAU = European Association of Urology; NMIBC = non-muscle-invasive bladder cancer.

One of 13 patients who had low/intermediate-risk NMIBC and who was frail (CFS ≥4) either died from bladder cancer or progressed. Similarly, only two (of 13) such patients had bladder cancer recurrence during follow-up (Table 3).

There was no significant difference between frail (CFS ≥4) and non-frail patients with regards to acute admissions related to the natural history of bladder cancer. However, frail patients had much higher complication rates following procedures for both procedure-related causes and other causes (Table 4).

**Table 4:** Complication rates comparing non-frail (CFS ≤ 3) and frail (CFS ≥ 4) patients

	Non-frail	Frail	*p-*value
Number, *n*	37	32	n/a
Higher risk NMIBC (%)	22 (59.5)	18 (56.3)	0.811
Bladder cancer acute admissions per patient, mean	0.73	0.41	0.479
Total procedures	398	137	n/a
30-day complications total (%)	33 (8.3)	35 (25.5)	**<0**.**05**
Procedure-related complications (%)	18 (4.5)	15 (10.9)	**<0**.**05**
Non-procedure-related complications (%)	15 (3.7)	20 (14.6)	**<0**.**05**

Higher risk NMIBC is defined as per EAU ‘high’ and ‘very high’ risk categories; statistical significance was measured using Fisher’s exact test. Bladder cancer acute complications expressed as the mean rate per patient and analysed with Student’s t test. Total procedures include both surveillance flexible cystoscopies and non-surveillance procedures. Thirty-day complication rates following a procedure are expressed as total, procedure-related causes and non-procedure-related causes which were analysed with Fisher’s exact test.

CFS = Rockwood Clinical Frailty Scale; EAU = European Association of Urology; NMIBC = non-muscle-invasive bladder cancer; n/a = median not defined.

## Discussion

These data show that there is an unsurprising decrease in OS with increasing CFS score, Charlson index and ASA grade. However, there does not seem to be a statistical difference in RFS or PFS in these groups. Expressed alternatively, there is no difference in the natural history of their NMIBC, but the different confounding patient factors around them significantly alter how we should approach management. We argue that frailer, more comorbid and higher anaesthetic risk patients should not have the same protocol-driven follow-up regime as their healthier counterparts. Such frail patients with a limited life expectancy may wish to prioritise quality of life by minimising unnecessary investigations and procedures.

Acute admissions due to bladder cancer during surveillance are similar between frail and non-frail patients, which again supports our hypothesis that the natural history of bladder cancer is similar between these groups. The proportion of higher risk NMIBC was similar between frailty groups. Furthermore, we found that complications after procedures were almost three times as high for frail patients. The causes of these complications are multifactorial; both procedure-related complications and other medical ailments brought such patients back into hospital within 30 days (Table 4). This will likely have had a negative impact on patient quality of life.

The causes of the complications were categorised by a consultant urologist and certain inferences were made based on the likelihood of a procedure-related cause. For example, there were nine trauma-related re-admissions, such as a fall within 30 days of a procedure, but these were deemed not related to the procedure itself. Notes were scrutinised to ensure that certain known complications of procedures such as urinary tract infections were not the cause in such cases. A detailed breakdown of the aetiology of non-procedure-related complications is elaborated on in Appendix 1 (available online). These include, but are not limited to, significant cardiovascular and respiratory events, which we believe are a consequence of competing comorbidity and poor physiological reserve.

All groups underwent broadly similar numbers of surveillance procedures (Table 2). This indicates that watchful waiting was not employed routinely. We noted some sporadic and inconsistent significance in the rates of surveillance and non-surveillance procedures between the different categories (Table 2), which could be due to the methodology. Many of the frail patients will have died shortly after diagnosis. Cystoscopic surveillance tends to be front-loaded to diagnose the more common early recurrences.^22^ However, this results in an overestimate in the rate of procedures when a patient dies early. This could explain the high rate of flexible cystoscopies in the CFS ≥ 5 group compared with the CFS = 4 group.

Very few patients who are both frail (CFS ≥ 4) and have a lower risk NMIBC (EAU low/intermediate) went on to have a recurrence or progression prior to death from other causes (Table 3). The one patient who progressed only did so from G2 low-grade to carcinoma in situ. This progression was managed with fulguration and surveillance thereafter before his subsequent death from metastatic lung carcinoma 12 months later. The remaining patient who recurred only had biopsies and fulguration on three separate recurrences, all of which were small, low grade and asymptomatic. We suggest that no patient in this potential watchful waiting group had a clinically significant progression.

There is an economic argument for trying to optimise the follow-up programme for frail patients. Age is one of the most significant risk factors for developing bladder cancer.^1^ Although the ≥75 years age group accounted for only 8.3% of the total UK population in 2018, this proportion is expected to increase to 10.6% in 2028, and 12.7% in 2038.^23^ This ageing population may increase the incidence of bladder cancers in the country leading to a significant healthcare and financial burden.

Non-cancer mortality has been shown to be significant for NMIBC patients aged >85 years regardless of risk category, and many such patients decline ongoing cystoscopic surveillance which supports an upper age limit for this.^24^ There are few retrospective studies on the impact of differing cystoscopic surveillance schedules for NMIBC patients.^25-27^ These draw different conclusions regarding the best way to time cystoscopies during follow-up, suggesting that this topic requires further research. Studies that advocate the current or even more intense protocol-driven follow-up do not explicitly compare patients of differing frailties.^26,27^ The strength of our study is the clear demonstration that frail patients do not have any differing oncological outcomes despite being more prone to dying from non-bladder cancer causes. For NMIBC diagnosed in older patients (aged 85 years), a high-intensity surveillance schedule does not appear to gain the same number of quality-adjusted life years compared with their younger counterparts (aged 65 years).^28^

### Study limitations

The frailty assessment was performed at the time of the primary TURBT when the cancer was risk-stratified and a decision made regarding follow-up. Further attempts to risk stratify any recurrences were not made explicitly. We accept that the risk profile of a NMIBC can change over the course of a patient’s life, which can alter a patient’s treatment and surveillance protocol. We identified patients retrospectively based on a flexible cystoscopy within 2 years prior to death. This method of patient identification may not have captured certain patients, including those who had since been upstaged to muscle-invasive bladder cancer and left the surveillance programme. Furthermore, patients who have since been discharged from the surveillance programme will not have been captured in our analysis. It follows that the combination of the above would introduce some bias against the most and least at-risk patient cohorts. When the cause of death was not conclusive from electronic hospital records (e.g. out-of-hospital deaths), a consultant urologist’s opinion was used. This opinion relied on the patient’s general condition prior to death and therefore inferences were made. Our study is limited by its small cohort size, as well as its retrospective nature and the lack of a prospective control group. However, the long follow-up duration for those with no significant frailty should minimise the bias towards underestimating progression with very low disease-specific mortality.

## Conclusion

The surveillance protocol for NMIBC currently does not factor in frailty, comorbidities or anaesthetic risk. We have shown that there is worsening OS for worsening CFS, Charlson and ASA categories despite similar PFS and RFS. Patients with higher scores are receiving broadly similar rates of surveillance cystoscopies and non-surveillance procedures that may not be required. These procedures lead to higher rates of complications and re-admissions for complications in the frail cohort.

Furthermore, we have identified no frail patients with a low/intermediate-risk index tumour who went on to have clinically significant disease progression. Patients who fall into this group could form a watchful waiting cohort in future management strategies.

Further prospective research into a lower-intensity follow-up protocol is needed. This can better balance the financial impact and morbidity of multiple care episodes against the detection of a clinically significant recurrence for individual patients, leading to a more personalised approach to bladder cancer surveillance.
